# Correlation of shear-wave elastography stiffness and apparent diffusion coefficient values with tumor characteristics in breast cancer

**DOI:** 10.1038/s41598-024-57832-2

**Published:** 2024-03-26

**Authors:** Mi-ri Kwon, Inyoung Youn, Eun Sook Ko, Seon-Hyeong Choi

**Affiliations:** 1 Department of Radiology, Kangbuk Samsung Hospital, Sungkyunkwan University School of Medicine, Seoul, South Korea; 2 Department of Radiology, Samsung Medical Center, Sungkyunkwan University School of Medicine, 81 Irwon-ro, Gangnam-gu, Seoul, 06351 South Korea; 3 Queen’s U Clinic, Seoul, South Korea

**Keywords:** Breast cancer, Cancer

## Abstract

We aimed to investigate the correlation between shear-wave elastography (SWE) and apparent diffusion coefficient (ADC) values in breast cancer and to identify the associated characteristics. We included 91 breast cancer patients who underwent SWE and breast MRI prior to surgery between January 2016 and November 2017. We measured the lesion’s mean (E_mean_) and maximum (E_max_) elasticities of SWE and ADC values. We evaluated the correlation between SWE, ADC values and tumor size. The mean SWE and ADC values were compared for categorical variable of the pathological/imaging characteristics. ADC values showed negative correlation with E_mean_ (r =  − 0.315, *p* = 0.002) and E_max_ (r =  − 0.326, *p* = 0.002). SWE was positively correlated with tumor size (r = 0.343–0.366, *p* < 0.001). A higher SWE value indicated a tendency towards a higher T stage (*p* < 0.001). Triple-negative breast cancer showed the highest SWE values (*p* = 0.02). SWE were significantly higher in breast cancers with posterior enhancement, vascularity, and washout kinetics (*p* < 0.02). SWE stiffness and ADC values were negatively correlated in breast cancer. SWE values correlated significantly with tumor size, and were higher in triple-negative subtype and aggressive imaging characteristics.

## Introduction

The incidence of breast cancer continues to increase, and it is the most commonly diagnosed cancer and the leading cause of death among women worldwide^1^. Mammography and breast ultrasonography (US) play essential roles in the diagnosis of breast cancer. Further, additional functional imaging techniques, including magnetic resonance imaging (MRI), have been integrated into the diagnostic process^2^. However, the biological features of tumors, such as the stiffness or cellularity of breast masses, cannot be assessed using conventional breast imaging modalities.

Elastography is an imaging modality based on tissue stiffness. The following two techniques, which differ in the type of stress applied, are widely used in breast imaging: strain elastography and shear-wave elastography (SWE)^3^. Unlike strain elastography, SWE is highly reproducible and can quantitatively measure tissue stiffness without operator dependency, providing more objective measurements that are more useful for determining tissue characteristics. Supplemental use of SWE with B-mode US improves diagnostic performance in differentiating malignant and benign breast lesions using different tissue stiffness^4,5^. Recent studies have reported that a larger tumor size and a higher histological grade are independently associated with a higher mean stiffness^6,7^. Moreover, triple-negative and human epidermal growth factor receptor 2 (HER2)-positive tumors exhibit higher stiffness than estrogen receptor (ER)-positive tumors^7^.

Diffusion-weighted imaging (DWI) is an advanced functional MRI technique that can provide tissue contrast without gadolinium contrast medium injections^2,8^. The apparent diffusion coefficient (ADC) calculated from DWI provides quantitative and qualitative information on tumor cellularity, integrity of the cell membrane, and microstructures using the Brownian motion of water molecules in the tissue^8,9^. DWI is useful for lesion detection, distinguishing between malignant and benign lesions, and assessing prognostic biomarkers of breast tumors^10,11^. Some studies have reported that ADC values are associated with tumor size, histological grade, and molecular subtype; however, this relationship remains debatable^12–14^.

We hypothesized a potential relationship between tumor stiffness and cellularity in breast cancer, along with potential associated characteristics. To the best of our knowledge, only a few studies have evaluated the relationship between SWE and ADC values^15,16^. Therefore, the objective of this study was to assess the correlation between quantitative elasticities of breast cancer, as indicated by SWE values, and cellularity measured by ADC values obtained through DWI. Additionally, we aimed to identify the pathological and imaging characteristics associated with SWE and ADC values in breast cancer.

## Results

### Patient characteristics

**Figure 1:**
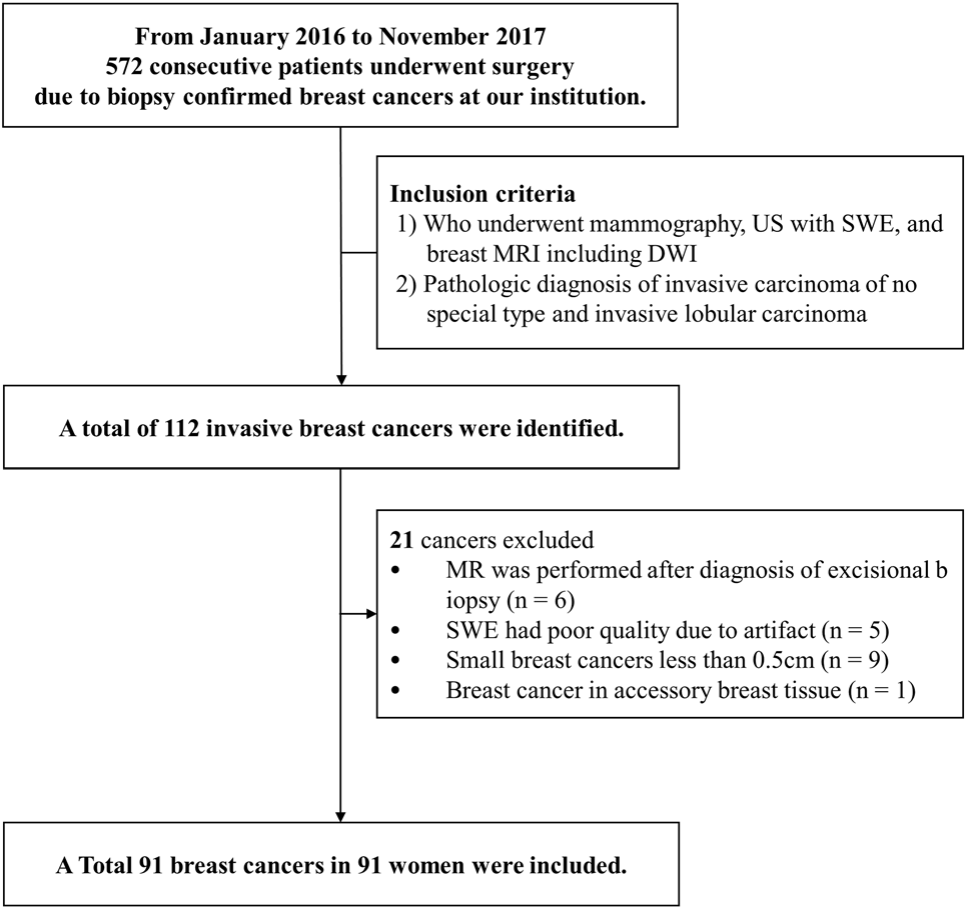
Flowchart of study population.

This study included a total of 91 women (mean age: 52.8 ± 12.2 years, range: 31–87 years) with 91 breast cancers (Fig. 1). The pathological tumor types were invasive carcinoma of no special type (n = 87), and invasive lobular carcinoma (n = 4). The mean size of tumors was 22.2 ± 14.2 mm. Among the 91 breast cancers, 83 (91.2%) were masses, and 8 (0.8%) were non-mass lesions on US and MRI. Table 1 summarizes the clinicopathological characteristics of patients.

**Table 1:** Clinicopathological characteristics of the 91 patients.

Characteristics	Total (n = 91)
Age (years)	52.8 ± 12.2
Pathologic type of tumor	
Invasive carcinoma of no special type	87 (95.6)
Invasive lobular carcinoma	4 (4.4)
T stage	
T1	49 (53.9)
T2	37 (40.7)
T3	5 (5.5)
Tumor size (mm)	22.2 ± 14.2
ER	
Negative	22 (24.2)
Positive	69 (75.8)
PR	
Negative	34 (37.4)
Positive	57 (62.6)
HER2	
Negative	66 (72.5)
Positive	25 (27.5)
Ki-67	
Low (< 20%)	66 (72.5)
High (≥ 20%)	25 (27.5)
Molecular subtype	
Luminal A	40 (44.0)
Luminal B	30 (33.0)
HER2-enriched	6 (6.6)
Triple-negative	15 (16.5)

Continuous variables are presented as mean ± standard deviation and categorical variables are presented as numbers of patients (percentages).

*ER* estrogen receptor, *HER2* human epidermal growth factor receptor 2, *PR* progesterone receptor.

### Correlation analysis of SWE and ADC values

**Figure 2:**
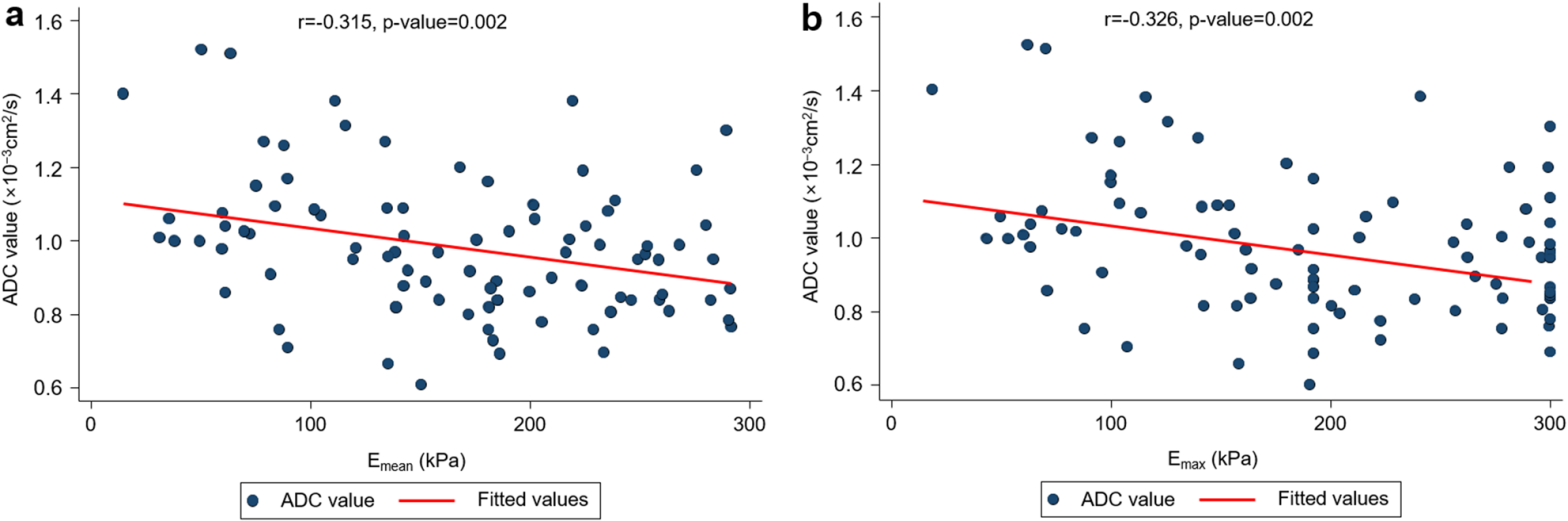
Scatter plot of shear wave elasticity and apparent diffusion coefficient values.

The mean values of mean elasticity (E_mean_) and maximum elasticity (E_max_) of SWE were 167.7 ± 75.0 kPa (range, 14.5–291.4 kPa) and 192.4 ± 84.8 kPa (range, 18.5–300 kPa). The mean ADC value of DWI was 0.982 ± 0.187 × 10^−3^ mm^2^/s (range, 0.610–1.520 × 10^−3^ mm^2^/s). There was a weak negative correlation between the E_mean_ and ADC values (r =  − 0.315, *p* = 0.002) and between the E_max_ and ADC values (r =  − 0.326, *p* = 0.002) (Fig. 2). There was a positive correlation between the SWE values and tumor size (E_mean,_ r = 0.343, *p* < 0.001; E_max_, r = 0.366, *p* < 0.001). There was no correlation between ADC values and tumor size (r =  − 0.045, *p* = 0.673).

### Comparison of SWE and ADC values according to pathologic and imaging characteristics

**Figure 3:**
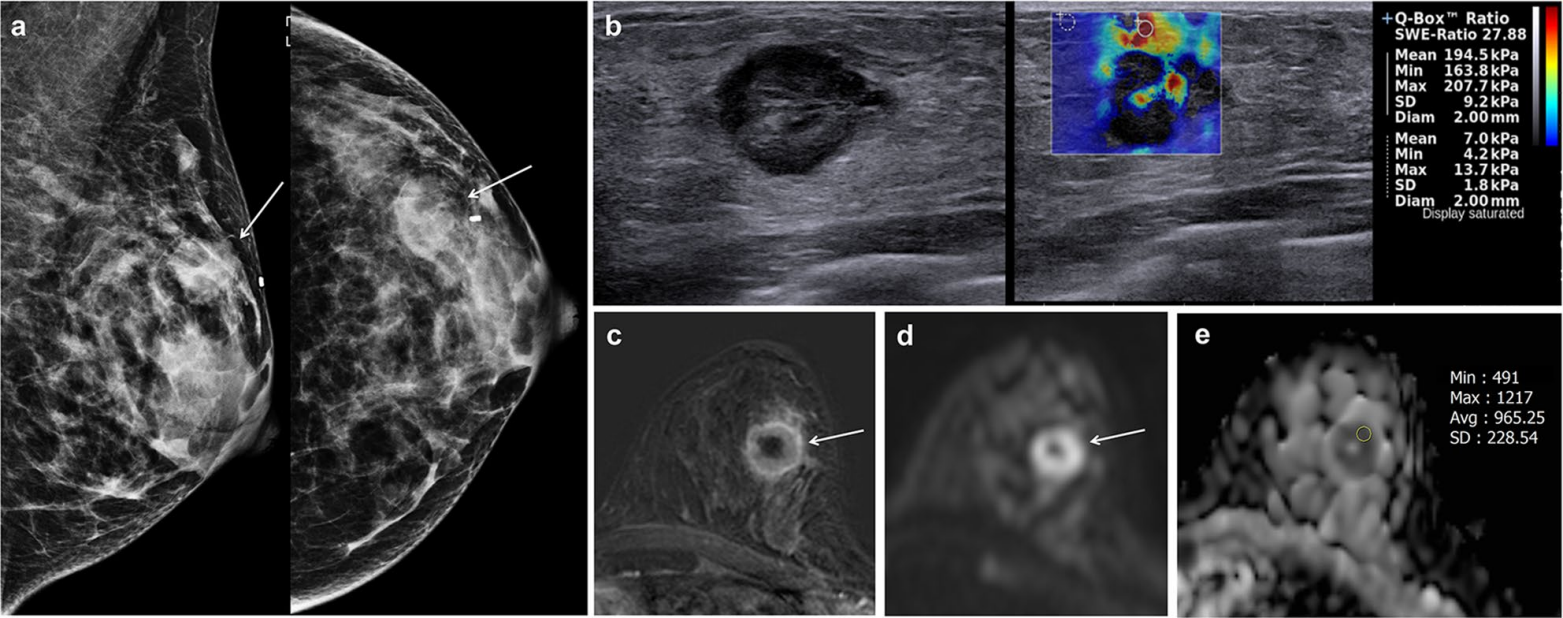
Imaging findings of triple-negative invasive breast cancer of no special type in the left breast of a 67-year-old woman. (**a**) Images obtained with mammography in the left mediolateral oblique (left) and left craniocaudal view (right) shows an irregular hyperdense mass (arrows) in the upper outer breast. (**b**) B-mode ultrasound image (left) shows a 22-mm microlobulated heterogenous mass with posterior enhancement. Shear-wave elastography (SWE, right) values are measured, with a mean elasticity of 194.5 kPa and a maximum elasticity of 207.7 kPa. (**c**) Axial contrast-enhanced T1-weighted subtraction magnetic resonance imaging (MRI) shows a round mass with rim enhancement (arrow). The mass shows early rapid and delayed washout enhancement. (**d**) Axial diffusion-weighted MRI (b value, 1000 s/mm^2^) demonstrates a mass with high signal intensity (arrow). (**e**) On the reconstructed apparent diffusion coefficient (ADC) map, the ADC value of the mass is 0.965 × 10^−3^ cm^2^/s. US-guided core-needle biopsy revealed invasive breast cancer of no special type that was estrogen receptor negative, progesterone receptor negative, and human epidermal growth factor receptor 2 negative.

**Figure 4:**
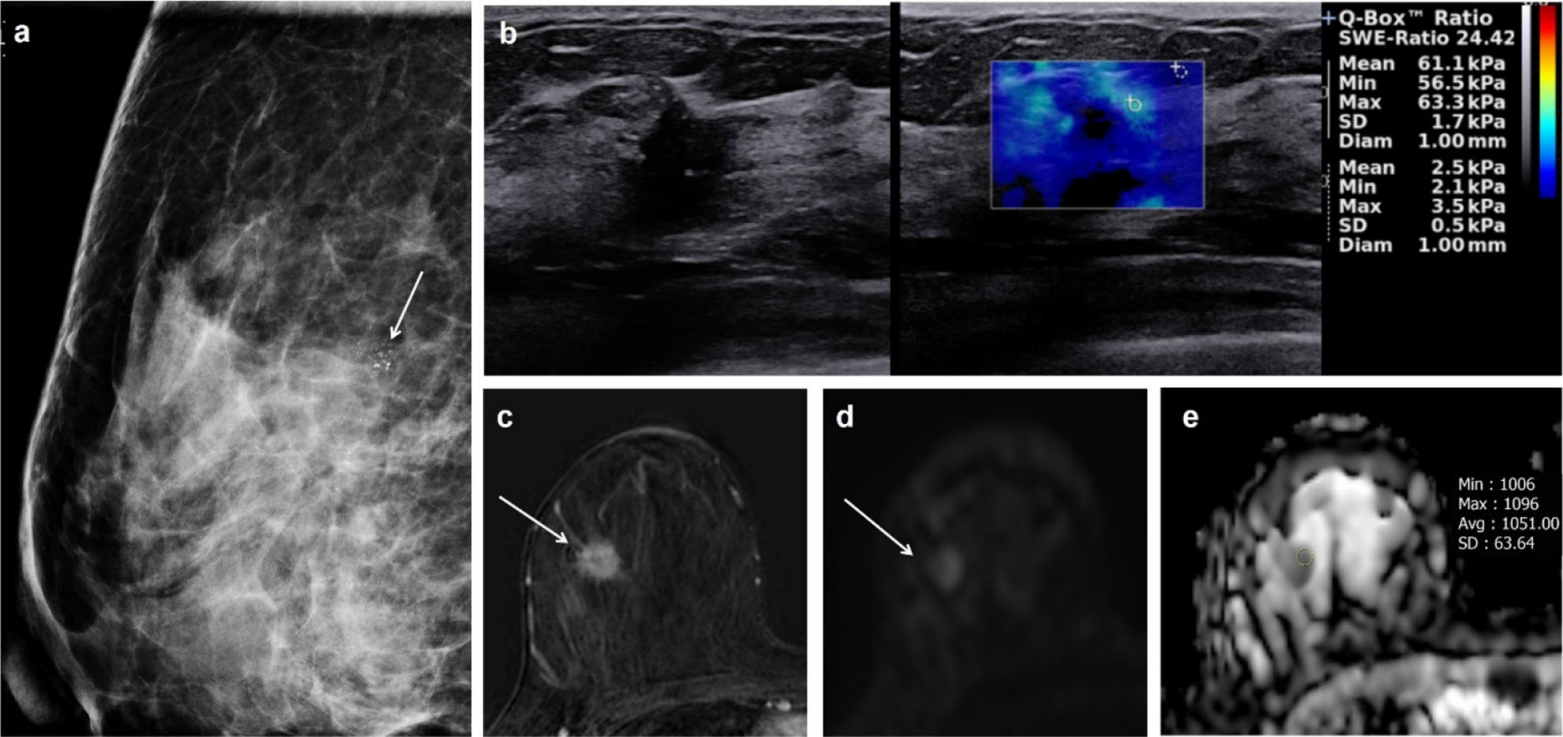
Imaging findings of a luminal A invasive breast cancer of no special type in the right breast of a 45-year-old woman. (**a**) Right magnification view shows suspicious microcalcifcations (arrow) in the upper outer quadrant. (**b**) B-mode ultrasound image (left) shows a 14-mm spiculated irregular hypoechoic mass containing calcifications. Shear-wave elastography (SWE, right) values are measured, with a mean elasticity of 61.1 kPa and a maximum elasticity of 63.3 kPa. (**c**) Axial contrast-enhanced T1-weighted subtraction magnetic resonance imaging (MRI) shows an irregular mass with heterogenous enhancement (arrow). The mass shows early rapid and delayed plateau enhancement. (**d**) Axial diffusion-weighted MRI (b value, 1000 s/mm^2^) shows a mass with high signal intensity (arrow). (**e**) On the reconstructed apparent diffusion coefficient (ADC) map, the ADC value of the mass is 1.048 × 10^−3^ cm^2^/s. US-guided core-needle biopsy revealed invasive breast cancer of no special type that was estrogen receptor positive, progesterone receptor positive, and human epidermal growth factor receptor 2 positive.

Table 2 summarizes the mean values and *p* values of the comparisons of E_mean_, E_max_, and ADC values according to the pathological characteristics. Higher SWE values were associated with a higher T stage (*p* < 0.001). The SWE values differed significantly according to molecular subtype (E_mean_, *p* = 0.02; E_max_, *p* = 0.02). Triple-negative breast cancer was the subtype with the highest E_mean_ and E_max_ values (202.48 ± 80.64 kPa and 231.23 ± 91.38 kPa, respectively), and luminal A breast cancer was the subtype with the lowest E_mean_ and E_max_ values (142.33 ± 72.07 kPa and 162.68 ± 79.55 kPa, respectively) (Figs. 3, 4).

**Table 2:** Elasticity and apparent diffusion coefficient values according to pathological characteristics.

	E_mean_	E_max_	ADC
Pathological type			
Invasive carcinoma of no special type (n = 87)	169.86 ± 74.91	194.6 ± 84.96	0.984 ± 0.19
Invasive lobular carcinoma (n = 4)	119.7 ± 67.95	143.78 ± 75.8	0.940 ± 0.118
*P* value	0.24	0.28	0.52
T stage			
T1 (n = 49)	146.37 ± 74.37	166.89 ± 82.52	0.999 ± 0.206
T2 (n = 37)	185.6 ± 69.38	214.78 ± 79.32	0.955 ± 0.171
T3 (n = 5)	243.46 ± 33.65	276.18 ± 47.3	1.017 ± 0.051
*P* value	0.003	0.002	0.51
*P* value for trend	< 0.001	< 0.001	0.54
Immunohistochemistry			
ER			
Negative (n = 22)	189.27 ± 81.0	215.41 ± 92.26	0.980 ± 0.150
Positive (n = 69)	160.77 ± 72.23	185.02 ± 81.68	0.980 ± 0.200
*P* value	0.15	0.18	0.95
PR			
Negative (n = 34)	174.53 ± 80.91	197.71 ± 92.89	0.994 ± 0.171
Positive (n = 57)	163.56 ± 71.65	189.17 ± 80.35	0.975 ± 0.198
*P* value	0.52	0.66	0.62
HER2			
Negative (n = 66)	161.83 ± 77.2	187.07 ± 87.53	0.976 ± 0.192
Positive (n = 25)	183.04 ± 67.89	206.34 ± 77.21	0.996 ± 0.175
*P* value	0.21	0.31	0.64
Ki-67			
Low (n = 66)	162.96 ± 73.08	186.31 ± 82.63	0.992 ± 0.205
High (n = 25)	180.04 ± 80.02	208.34 ± 90.2	0.954 ± 0.126
*P* value	0.36	0.29	0.39
Molecular subtype			
Luminal A (n = 40)	142.33 ± 72.07	162.68 ± 79.55	1.013 ± 0.206
Luminal B (n = 30)	183.84 ± 65.57	212.82 ± 75.94	0.953 ± 0.189
HER2-enriched (n = 6)	168.5 ± 84.98	190.82 ± 96.35	1.024 ± 0.107
Triple-negative (n = 15)	202.48 ± 80.64	231.23 ± 91.38	0.940 ± 0.144
*P* value	0.02	0.02	0.10

Variables are presented as mean ± standard deviation.

*ADC* apparent diffusion coefficient, *E*_*mean*_ mean elasticity, *E*_*max*_ maximum elasticity, *ER* estrogen receptor, *HER2* human epidermal growth factor receptor 2, *PR* progesterone receptor.

Among the US characteristics, the SWE values were significantly higher in breast cancers with vascularity compared to those without vascularity (*p* = 0.001) (Table 3). Breast masses with posterior enhancement or combined patterns showed higher SWE values (E_mean_, *p* = 0.02; E_max_, *p* = 0.03). Among the MRI characteristics, the SWE values were higher in breast cancer showing washout kinetics on the delayed phase and lower in breast cancer showing persistent kinetics (E_mean_, *p* = 0.01; E_max_, *p* = 0.009) (Table 4). Breast masses with an irregular shape and not circumscribed margin had lower ADC values (*p* = 0.03 and *p* = 0.02, respectively). There were no significant differences in SWE and ADC values according to lesion type (mass or non-mass lesion) or other imaging characteristics.

**Table 3:** Elasticity and apparent diffusion coefficient values according to ultrasound characteristics.

	E_mean_	E_max_	ADC
US finding			
Mass (n = 83)	170.59 ± 76.23	196.07 ± 85.93	0.984 ± 0.179
Non-mass lesion (n = 8)	132.44 ± 49.00	147.84 ± 57.58	0.957 ± 0.280
*P* value	0.20	0.15	0.72
Mass (n = 83)			
Shape			
Oval (n = 20)	138.40 ± 77.29	161.73 ± 87.27	1.063 ± 0.169
Round (n = 13)	187.82 ± 72.98	223.18 ± 80.42	0.952 ± 0.207
Irregular (n = 50)	179.63 ± 74.12	203.45 ± 84.02	0.959 ± 0.169
*P* value	0.08	0.08	0.06
Margin			
Circumscribed (n = 9)	170.07 ± 85.49	199.94 ± 95.67	0.993 ± 0.182
Not circumscribed (n = 74)	170.65 ± 75.68	195.61 ± 85.39	0.983 ± 0.18
*P* value	0.98	0.89	0.88
Orientation			
Parellel (n = 66)	171.05 ± 73.41	198.11 ± 82.70	0.99 ± 0.184
Not parellel (n = 17)	168.79 ± 88.94	188.06 ± 100.05	0.961 ± 0.161
*P* value	0.91	0.67	0.55
Echo pattern			
Heterogenous (n = 36)	186.42 ± 67.62	211.06 ± 76.83	1.018 ± 0.175
Hypoechoic (n = 47)	158.72 ± 80.75	184.84 ± 91.35	0.959 ± 0.18
*P* value	0.10	0.17	0.14
Posterior features			
No (n = 20)	141.44 ± 73.57	166.07 ± 88.35	1.011 ± 0.194
Shadowing (n = 26)	157.44 ± 75.14	181.22 ± 86.58	0.972 ± 0.201
Enhancement and combined (n = 37)	194.93 ± 72.47	222.03 ± 78.47	0.978 ± 0.158
*P* value	0.02	0.03	0.75
Non-mass lesion (n = 8)			
Distribution			
Non-segmental (n = 5)	133.22 ± 50.91	146.82 ± 54.85	0.988 ± 0.326
Segmental (n = 3)	130.50 ± 63.50	150.40 ± 88.53	0.881 ± 0.173
*P* value	0.95	0.95	0.69
Echo pattern			
Heterogenous (n = 4)	149.25 ± 36.29	169.15 ± 43.45	0.903 ± 0.178
Hypoechoic (n = 4)	110.03 ± 62.54	119.43 ± 70.68	1.03 ± 0.417
*P* value	0.34	0.30	0.601
Posterior shadowing			
No (n = 4)	108.13 ± 59.30	123.63 ± 77.90	1.091 ± 0.383
Yes (n = 4)	150.68 ± 37.74	166.00 ± 39.49	0.857 ± 0.167
*P* value	0.29	0.38	0.32
Calcifications			
Absent (n = 70)	165.34 ± 77.95)	188.64 ± 86.53	0.996 ± 0.182
Present (n = 21)	175.38 ± 65.27)	204.78 ± 79.67	0.935 ± 0.2
*P* value	0.59	0.45	0.19
Vascularity			
Absent (n = 33)	135.91 ± 75.77	156.48 ± 86.09	1.015 ± 0.195
Present (n = 56)	187.57 ± 68.75	215.38 ± 77.18	0.969 ± 0.181
*P* value	0.001	0.001	0.26

Variables are presented as mean ± standard deviation.

*ADC* apparent diffusion coefficient, *E*_*mean*_ mean elasticity, *E*_*max*_ maximum elasticity, *US* ultrasonography.

**Table 4:** Elasticity and apparent diffusion coefficient values according to MRI characteristics.

	E_mean_	E_max_	ADC
MRI finding			
Mass (n = 83)	170.01 ± 76.32	195.03 ± 86.11	0.989 ± 0.188
Non-mass enhancement (n = 8)	143.21 ± 57.66	164.66 ± 68.82	0.911 ± 0.163
*P* value	0.34	0.34	0.27
Mass (n = 83)			
Shape			
Oval or round (n = 26)	154.55 ± 80.18	178.68 ± 89.59	1.057 ± 0.216
Irregular (n = 57)	177.06 ± 74.14	202.49 ± 84.21	0.958 ± 0.172
*P* value	0.22	0.25	0.03
Margin			
Circumscribed (n = 16)	149.37 ± 75.82	170.75 ± 84.58	1.083 ± 0.225
Not circumscribed (n = 67)	174.94 ± 76.17	200.83 ± 86.07	0.966 ± 0.173
*P* value	0.23	0.21	0.02
Internal enhancement			
Homogenous (n = 10)	169.07 ± 71.50	187.45 ± 78.94	1.062 ± 0.153
Heterogenous (n = 66)	169.30 ± 76.52	194.91 ± 86.36	0.968 ± 0.18
Rim enhancement (n = 7)	178.03 ± 91.72	207.07 ± 104.53	1.078 ± 0.274
*P* value	0.96	0.90	0.14
Non-mass enhancement (n = 8)			
Distribution			
Non-segmental (n = 4)	124.15 ± 45.68	140.82 ± 42.25	0.94 ± 0.217
Segmental (n = 4)	162.28 ± 68.58	188.50 ± 88.03	0.883 ± 0.113
*P* value	0.39	0.37	0.66
Internal enhancement			
Heterogenous (n = 4)	122.53 ± 42.97	146.07 ± 51.11	0.972 ± 0.213
Clumped (n = 4)	163.90 ± 69.07	183.25 ± 86.70	0.851 ± 0.081
*P* value	0.35	0.49	0.33
Initial phase kinetics			
Medium (n = 14)	149.70 ± 73.52	163.29 ± 74.10	0.962 ± 0.191
Rapid (n = 77)	170.92 ± 75.26	197.65 ± 86.03	0.985 ± 0.187
*P* value	0.33	0.16	0.67
Delayed phase kinetics			
Persistent (n = 21)	127.18 ± 70.20	145.83 ± 78.20	1.025 ± 0.216
Plateau (n = 24)	169.32 ± 79.92	192.73 ± 89.47	1.003 ± 0.172
Washout (n = 46)	185.27 ± 68.62	213.42 ± 78.25	0.952 ± 0.179
*P* value	0.01	0.009	0.27

Variables are presented as mean ± standard deviation.

*ADC* apparent diffusion coefficient, *E*_*mean*_ mean elasticity, *E*_*max*_ maximum elasticity, *MRI* magnetic resonance imaging, *US* ultrasonography.

## Discussion

Since the introduction of SWE and DWI, various clinical applications have been explored. In this study, we investigated the relationship between elasticity and ADC values in breast cancer and found a negative correlation between them. SWE can provide information on tissue stiffness by quantitatively measuring the real-time stiffness of tissue superimposed on a B-mode image quantitatively^17^. SWE measurement increases in many specific cases, such as in solid tumors as a pathological process or fibrosis as a physiological process^18^. Tumor stiffness is determined by several factors, including fibrosis, cellularity, and necrosis^19^. Significant collagen deposition, linearization, and bundling lead to stiffening and remodeling of extracellular matrix, which corresponds to the malignant transition to invasive cancer^20,21^_._ These alternations are induced by hypoxia, a fundamental biological feature associated with angiogenesis and tumor stiffness, which compromises antitumor immunity and potentiates tumor cell growth, leading to cancer growth, metastasis, and resistance to treatment^22^. These changes affect SWE values in breast cancer. ADC values also reflect tumor microstructures, including tumor cellularity, fluid viscosity, membrane permeability, and extracellular matrix stiffness, which drive fibrosis to stiffening of the stroma^14^. Additionally, low ADC values, indicating high cellularity, lead to increased hypoxia and interstitial hypertension, and finally increased microenvironment-associated metastasis^23^.

Matsubayashi et al.^16^ reported that US elastographic strain score and MRI diffusion were significantly correlated with fibrotic changes in breast disease based on pathologic examination. They classified the elastographic strain score as 1–5 points according to the strain map pattern, whereas we measured the objective SWE (E_mean_ and E_max_) of breast cancers. Recently, Orguc et al.^15^ reported that the SWE and ADC values were correlated in 147 benign and malignant breast lesions. In contrast, we focused on breast malignancies to evaluate the correlation between SWE and ADC values, which may be affected by microstructural changes in malignancies. In addition, we compared the SWE and ADC values according to the imaging characteristics of gray-scale US and DCE-MRI. Our results demonstrated a weak negative correlation between stiffness measured using SWE and ADC values in breast cancers. The pathological explanation of this correlation could be explained by tumor cellularity, degree of stromal fibrosis, extracellular matrix stiffness, or tumor to stroma ratio in breast cancer influencing the SWE and ADC values; these microstructural changes in breast cancer result in a correlation between these values. In addition, tumor stiffness may correspond to diffusion restrictions in breast cancer.

Our results indicated that SWE values were significantly higher in breast cancers with posterior enhancement, vascularity, and washout kinetics. Posterior enhancement is the phenomenon that sound transmission is unimpeded in its passage through the mass^24^. It is well known that high cellularity or tumor necrosis is associated with posterior enhancement and identified in 24–41% of triple-negative breast cancers^25^. Additionally, high-grade breast cancer is linked to higher chance of posterior enhancement^26^. Vascularity and enhancement kinetics reflect angiogenesis, which plays important role in tumor growth and progression and is mediated by the tumor microenvironment, including extracellular matrix stiffening and mechanical forces^27^. Among the enhancement kinetics, washout kinetics on the delayed phase are associated with poorer clinical outcomes^28^. Our findings suggest that imaging characteristics associated with aggressive biology and poor clinical outcomes are also related to higher elasticity. Meanwhile, the ADC values were lower in breast masses with irregular shape and not circumscribed margin. Larger studies are required to validate our findings.

Using SWE, the mean E_mean_ value of the breast cancers in our study was determined to be 167.7 ± 75.0 kPa, which is comparable to the range of 133–153 kPa reported in previous studies, and higher than the cutoff values of 72–100 kPa used to for distinguishing between malignant and benign lesions^4,29^. Our study showed higher SWE values for higher T stages/larger tumor sizes, which are poor prognostic factors, consistent with previous studies^6,7,30^. Triple-negative breast cancer had the highest E_mean_ and E_max_ values, whereas luminal A breast cancer had the lowest E_mean_ and E_max_ values. Biophysical and biochemical assessments revealed that extracellular matrix stiffness, immune infiltrate, and tumor progression differed according to tumor subtype. Triple-negative cancers have poor clinical outcomes and are associated with aggressive histology^31^. Triple-negative cancers have yielded controversial results regarding elasticity. Several previous studies have found that triple-negative cancers and ER/progesterone receptor (PR) negativity are correlated with higher SWE values because they have higher heterogeneous extracellular stiffness, which reflects an increased number of infiltrating immune cells and macrophages, more linearized collagen, and invasion signaling, which is consistent with our results^7,30,32^, whereas others have reported that triple-negative cancers are less stiff than other breast cancers^33^.

The ADC value from DWI reflects cellular density and quantifies water diffusion, and its major strength is that it provides a quantitative measure of the observed diffusion restriction. We found a mean ADC value of 0.982 × 10^−3^ mm^2^/s for breast cancers and a maximum value of 1.520 × 10^−3^ mm^2^/s, which is comparable to the previously reported ADC cutoff values of 1.1–1.6 × 10^−3^ mm^2^/s for distinguishing between benign and malignant lesions^34,35^. Several studies have investigated the relationship between ADC values and pathological characteristics and have shown conflicting results. Some studies have found that lower ADC values are associated with larger tumor size, higher histological grade, and invasiveness^36–38^, whereas others have found no significant relationship^12,14,39^. Several studies showed that HER2-enriched tumors have higher ADC values, whereas ER-positive tumors have lower ADC values than ER-negative tumors^11^. However, a recent meta-analysis found that the ADC cannot discriminate between molecular subtypes, which suggests that the ADC cannot be used as a surrogate marker for disease stage or proliferation activity^40^. In our study, there were no significant differences between the ADC values and different pathological types, T stages, or molecular subtypes. These results may be attributed to relatively small sample sizes.

Our study had several limitations. First, it was a retrospective study conducted at a single institution with a relatively small number of breast cancer cases. Further, we included patients who underwent both SWE and DWI before breast cancer surgery, which might have caused a selection bias. Second, breast cancers are heterogeneous in stiffness and cellularity, and the precise areas measured using SWE and DWI are likely to differ. However, it was inevitable to acquire same areas of the tumor because the modalities were different. To overcome these issues, we attempted to acquire the most representative part of the tumor by measuring SWE and ADC in the area with the largest diameter. Third, we did not evaluate the inter-observer agreement of SWE and ADC values. Fourth, although SWE was performed using the same US machine by a single radiologist to avoid interoperator variability, patient-related or clinical factors might have influenced the quality of the images and SWE values. Fourth, the range of SWE values were higher than what has been reported in previous articles^6,7,30,32^, and some cancer cases exhibited value of 300 kPa, which might exceed the established limit. Lastly, we did not correlate SWE or ADC values with histopathological findings such as collagen, extracellular matrix, or the degree of stromal fibrosis in breast cancers, which could help explain the probable cause of the relationship between SWE and ADC values. However, the purpose of our study was not to determine whether there is an exact point-to-point correlation between imaging and pathological findings but to determine the correlation between the two values measured by different modalities in breast cancers. Larger dedicated studies analyzing pathological correlations are necessary to validate our findings.

In conclusion, SWE stiffness and ADC values were negatively correlated in breast cancer. SWE values significantly correlated with tumor size and were higher in triple-negative subtypes and imaging characteristics associated with aggressive biology. Our study suggests a potential relationship between SWE stiffness and ADC values, reflecting tumor microenvironment and highlights the potential utility of SWE as an image biomarker for identifying aggressive tumor biophysical properties.

## Methods

### Study population

This retrospective study was approved by the Institutional Review Board of Kangbuk Samsung Hospital (Approval No. KBSMC 2021-08-051), and the requirement for written informed consent was waived. This study was performed in accordance with relevant guidelines and regulations. Between January 2016 and November 2017, 572 consecutive patients underwent surgery for biopsy-confirmed breast cancer at our institution. We included patients who underwent US with SWE and breast MRI, including DWI, for invasive breast cancer, limited to invasive carcinoma of no special type or invasive lobular carcinoma, resulting in 112 patients with invasive breast cancer. Among them, we excluded patients for who underwent MRI after diagnosis through excisional biopsy or vacuum-assisted biopsy (*n* = 6), patients with poor-quality SWE due to artifacts (*n* = 5), small cancers (less than 0.5 cm; *n* = 9), and breast cancer in accessory breast tissue (*n* = 1). Finally, this study included 91 breast cancers in 91 women (Fig. 1).

### Ultrasound and shear-wave elastography imaging

Real-time grayscale breast US imaging was performed using a 4–15 MHz linear transducer (Aixplorer; SuperSonic Imagine, Aix-en-Provence, France) for breast lesions in two orthogonal planes by one of four board-certified faculty radiologists with 5–25 years of experience in breast imaging. In addition, color Doppler study was performed for breast lesions. After that, SWE images were generated without pressure induced by the transducer by a single radiologist (S.H.C.) with eight years of experience in elastography on the same day. The B-mode semitransparent color map revealed stiffness values ranging from dark blue to red (0–180 kPa) and the quantitative measurement scale was set at a maximum of 300 kPa. Elasticity values were measured in the plane with the longest diameter of each lesion. Quantitative elasticity values were measured using the system quantification tool, known as the “Q-Box,” which defined a diameter of 1 − 2 mm region of interest (ROI) that was positioned over the stiffest part of the lesion or surrounding tissue on the SWE image. The E_mean_ and E_max_ were recorded.

### Breast MRI and apparent diffusion coefficient values on diffusion-weighted imaging

Breast MRI was performed with the patient in a prone position using a 3 T system (Achieva, Philips Medical System, Best, Netherlands) with a dedicated seven-channel SENSE breast coil. After obtaining the localizer images, the following images were obtained: (1) axial non-contrast T2- and T1-weighted images; (2) DWI; (3) fat-suppressed dynamic contrast-enhanced (DCE) T1-weighted images at 1, 2, 3, 5, and 7 min after intravenous injection of a 0.1 mmol/kg bolus of gadobutrol (Gadovist, Bayer Schering Pharma, Berlin, Germany); and (4) delayed axial T1-weighted images. Before contrast agent injection, DWI with echo planar imaging was performed using the following scanning parameters: TR/TE, 3265/55; slice thickness, 4 mm; matrix size, 108 × 98; and field of view, 35 cm. DWI was performed with two b-values (0 and 1000 s/mm^2^), and ADC maps were automatically generated on a voxel-by-voxel basis before enhancement.

To measure the ADC value, two radiologists (M.K. and I.Y.) with 5 and 11 years of experience in breast imaging, respectively, determined the ROI by consensus. Similar to SWE, a circular ROI with a diameter of 1 − 2 mm was manually drawn on the slice on which the cancer showed the greatest diameter. The ROIs matched the solid-enhancing regions on DCE images and avoided areas of necrosis or cysts. The mean ADC values were recorded from DWI according to the recent European Society of Breast Imaging breast DWI guideline^10^.

### Data collection

We retrospectively reviewed the pathology reports of the final histopathological specimens and recorded the pathological type of the tumor, T stage, tumor size, ER, PR, HER2, and the Ki-67 index. The criterion for ER and PR positivity was the staining of ≥ 1% of the nuclei. Immunohistochemical HER2 scores of 3+ were classified as positive. Silver-enhanced in situ hybridization was used in cases with a HER2 score of 2+^41^. The cutoff expression level of Ki-67 was established at 20%^42^. Breast cancer molecular subtypes were classified as luminal A (ER+ and/or PR+, HER2−, Ki-67 < 20%), luminal B (ER+ and/or PR+, HER2−, Ki-67 ≥ 20%; ER+, HER2+, and any Ki-67), HER2-enriched (ER−, PR−, and HER2+), and triple-negative (ER−, PR−, and HER2−).

The imaging characteristics of breast US and MRI were assessed through a retrospective review by two radiologists in consensus (M.K. and I.Y.). They recorded the imaging characteristics of US, which were evaluated as follows: lesion type (mass or non-mass lesion), calcifications (absent or present), and vascularity (absent or present [internal vascularity or vessels in rim]) on color Doppler study^24^. For lesion types, a mass was defined as a space-occupying lesion depicted in two different projections^24^ and a non-mass lesion was defined as a discrete identifiable area of altered echotexture compared with to surrounding breast tissue that did not conform to a mass shape^43^. Further image findings were evaluated as follows^43^; for masses, shape (oval, round, or irregular), margin (circumscribed or not circumscribed), echo pattern (isoechoic, heterogeneous, or hypoechoic), orientation (parallel or not parallel), and posterior features (none, shadowing, or enhancement) according to BI-RADS US-lexicon^24^; for non-mass lesions, distribution (non-segmental or segmental), echo pattern (isoechoic, heterogeneous, or hypoechoic), and posterior shadowing (absent or present)^44^. According to BI-RADS MR-lexicon^24^, imaging characteristics of DCE-MRI were evaluated as follows: lesion type (mass or non-mass enhancement), enhancement kinetics at the initial phase (slow, medium, or rapid) and the delayed phase (persistent, plateau, or washout); shape (oval, round, or irregular), margin (circumscribed or not circumscribed), and internal enhancement (homogeneous, heterogeneous, or rim enhancement) for mass; distribution (non-segmental or segmental) and internal enhancement (homogeneous, heterogeneous, clumped, or clustered ring) for non-mass enhancement.

### Statistical analysis

Continuous variables were reported as the mean ± standard deviation (SD), and categorical variables were reported as the percentage and frequency. Correlation analysis using Pearson’s correlation coefficients was used to evaluate the correlation between E_mean_ (kPa) and E_max_ (kPa) on SWE, ADC values on DWI, and pathological tumor size (mm). Scatter plots were drawn to determine the correlation between the E_mean_, E_max_, and ADC values.

The mean ± SD values of the E_mean_, E_max_, and ADC were evaluated and compared according to each categorical variable of the pathological and imaging characteristics using independent sample t-tests and analysis of variance.

All statistical analyses were performed using the SPSS version 24 for Windows (IBM Corp., Armonk, NY, USA). Statistical significance was defined as *p* < 0.05.

## Data Availability

The datasets generated during and/or analysed during the current study are available from the corresponding author on reasonable request.
